# Partial Exactness for the Penalty Function of Biconvex Programming

**DOI:** 10.3390/e23020132

**Published:** 2021-01-21

**Authors:** Min Jiang, Zhiqing Meng, Rui Shen

**Affiliations:** 1 School of Management, Zhejiang University of Technology, Hangzhou 310023, China; jiangmin@zjut.edu.cn; 2 School of Economics, Zhejiang University of Technology, Hangzhou 310023, China; shenrui@zjut.edu.cn

**Keywords:** biconvex programming, partial optimum, partially exact penalty function, partial exactness, partial local stability

## Abstract

Biconvex programming (or inequality constrained biconvex optimization) is an important model in solving many engineering optimization problems in areas like machine learning and signal and information processing. In this paper, the partial exactness of the partial optimum for the penalty function of biconvex programming is studied. The penalty function is partially exact if the partial Karush–Kuhn–Tucker (KKT) condition is true. The sufficient and necessary partially local stability condition used to determine whether the penalty function is partially exact for a partial optimum solution is also proven. Based on the penalty function, an algorithm is presented for finding a partial optimum solution to an inequality constrained biconvex optimization, and its convergence is proven under some conditions.

## 1. Introduction

Multi-convex programming is a non-convex optimization problem [1,2], where biconvex programming is a special case. It is ubiquitous nowadays in fields such as control [3,4,5], machine learning [6,7], signal and information processing [8,9], communication [10,11], and also NP-hard problems [12]. The existing research on multi-convex programming mainly solves some very special models [12,13,14,15,16,17,18,19]. These studies all give specific methods for each special model. When problems change, new methods need to be studied. Biconvex programming is a simple case of multi-convex programming, and it is also studied in research [14] as a special case. Therefore, it is of great significance to study the algorithm of biconvex programming for solving practical engineering optimization problems.

Bilinear programming is the simplest case of biconvex programming and is the earliest and most studied as per [14]. There are two main ways to solve bilinear programming. One is the simplex algorithm based on sub-problems, and the other is the alternating direction method. For example, Liang and Bai [15] and Hajinezhad and Shi [16] both proposed the alternating direction method of multipliers (ADMM) algorithm for two special bilinear programming problems, where the extended Lagrangian penalty function uses a square penalty. Furthermore, Charkhgard et al. [17] presented a multi-linear programming algorithm using the linear programming algorithm.

In 2007, Gorski et al. [14] reviewed the development of the theory and algorithms of biconvex optimization. The biconvex functions have some good properties that are similar to convex functions, such as the biconvex separation theorem and an equivalence between local optimal solutions and stationary points. The biconvex programming algorithm is based on the idea that the solution to alternating sub-problems can converge to the stationary point of the original problem. For example, in 2015, Li et al. [18] studied an alternating convex search method to solve a stationary point problem of biconvex programming. In 2016, Shah et al. [19] presented an alternating search method with a square penalty function to solve biconvex programming, which had great effect in image recognition. Al-Khayyaltt and Falk [20] discussed an algorithm of biconvex programming using the idea of branch-and-bound.

In short, based on the above, the mainstream method used to solve biconvex programming is an alternative to subproblem solving, because the bi-convexity of biconvex programming guarantees the convergence of alternative search methods and effectually cuts down the scale of problem solving via decomposition calculation. Hence, in view of the large scale of biconvex programming(BCP), alternative subproblem solving will be the main method adopted in further research regarding biconvex programming.

In this paper we consider biconvex programming with inequality constraints as follows:

$$\begin{array}{ccc} \left(\mathrm{BCP}\right)\; & \min\; & h(u_{1},u_{2})\\ & s.t.\; & g_{i}\left(u_{1},u_{2}\right)\leq 0,\;i=1,2,\cdots ,m, \end{array}$$

where $h,g_{i}:R^{n_{1}}\times R^{n_{2}}\to R\left(i=1,2,\cdots ,m\right)$ are biconvex if $h(u_{1},u_{2})$ and $g_{i}\left(u_{1},u_{2}\right)$ are convex in $u_{1}\in R^{n_{1}}$ for every fixed $u_{2}$ and in $u_{2}\in R^{n_{2}}$ for every fixed $u_{1}$. Let $u=(u_{1},u_{2})$ and I={1,2,⋯,m}. The feasible set of (BCP) is denoted by:

$$U=\{\left(u_{1},u_{2}\right)\in R^{n_{1}}\times R^{n_{2}}|g_{i}\left(u_{1},u_{2}\right)\leq 0,\;i\in I\}.$$

Let

$$U\left(u_{2}\right)=\left\{u_{1}\in R^{n_{1}}|g_{i}\left(u_{1},u_{2}\right)\leq 0,\;i\in I\right\}.$$

When $u_{2}$ is fixed, define the suboptimal problem:

$$\begin{array}{ccc} \left(\mathrm{BCP}\right)\left(u_{2}\right)\; & \min\; & h(u_{1},u_{2})\\ & s.t.\; & u_{1}\in U\left(u_{2}\right). \end{array}$$

Let

$$U\left(u_{1}\right)=\left\{u_{2}\in R^{n_{2}}|g_{i}\left(u_{1},u_{2}\right)\leq 0,\;i\in I\right\}.$$

When $u_{1}$ is fixed, define the suboptimal problem:

$$\begin{array}{ccc} \left(\mathrm{BCP}\right)\left(u_{1}\right)\; & \min\; & h(u_{1},u_{2})\\ & s.t.\; & u_{2}\in U\left(u_{1}\right). \end{array}$$

Many practical engineering optimization problems can be transformed into biconvex programming, for example the two-cardinality sparse convex optimization problem and the nonconvex quadratic programming problem.

It is well known that an effective method to solve constrained optimization is by penalty function [21]. Its main idea is to transform a constrained optimization problem into a sequence of unconstrained optimization subproblems that are easier to solve. When the penalty function is not exact, many calculations are needed to solve unconstrained optimization subproblems so as to obtain an approximate solution to inequality constrained optimization. For example, it is proven that the exact penalty function method for solving constrained optimization is very efficient, as first proposed by Zangwill (1967) in [22]. In theory, if the penalty function is exact, to obtain an optimal solution to a constrained optimization problem only one unconstrained optimization subproblem is solved. Hence, the exact penalty function algorithm takes less time than the inexact penalty function algorithm. Additionally, this approach has recently received great attention from both theoretical and practical arenas. Many studies were later presented based on the exact penalty function algorithm, such as Rosenberg (1986) [23] and Di Pillo (1986) [24]. In addition, it is essential to determine the exactness of a penalty function under the stability condition [25,26]. Hence, this paper mainly focuses on the relationship between the partial exactness of the penalty function and the partial stability of biconvex programming. On the other hand, there exist other approaches to reduce constrained optimization problems to unconstrained ones; for example, the index method presented in the monograph [27] or the method for computable boundaries presented in [28]. This shows the significance of the study of the partial exact penalty function.

In order to ensure exactness, we propose the following penalty function:

$$H_{p}\left(u_{1},u_{2},\rho \right)=h\left(u_{1},u_{2}\right)+\rho \sum_{i\in I}\max{\left\{g_{i}\left(u_{1},u_{2}\right),0\right\}}^{p},$$

where penalty parameter ρ>0 and 0<p≤1. By the definition of exactness in [29], a penalty function is exact at p=0.5, but is not exact at p=1, such that $\min_{x\in R^{1}}f\left(x\right)=x\;s.t.\;x^{2}\leq 0$. Hence, the exactness of an optimization problem depends on the structure of the problem. In this paper, we will study the exactness of a more extensive penalty function for biconvex programming than the one presented in [30].

The remainder of the paper is organized as follows. In Section 2, for a partial optimum solution, the partial exactness of the penalty function is proven under the partial Karush–Kuhn–Tucker (KKT) condition or the partial stableness condition. An algorithm is presented to find out a partial optimum solution to (BCP) with convergence.

## 2. Partial Exactness and a Penalty Function for (BCP)

According to Gorski et al. [14], defining the partial optimum of (BCP) is very meaningful. The concept of the partial optimum of (BCP) is given as follows:

**Definition** **1.** Let $(u_{1}^{*},u_{2}^{*})\in U$. If:

(1) $$\begin{array}{c} h\left(u_{1}^{*},u_{2}^{*}\right)\leq h\left(u_{1},u_{2}^{*}\right),\forall u_{1}\in U\left(u_{2}^{*}\right), \end{array}$$

(2) $$\begin{array}{c} h\left(u_{1}^{*},u_{2}^{*}\right)\leq h\left(u_{1}^{*},u_{2}\right),\forall u_{2}\in U\left(u_{1}^{*}\right), \end{array}$$

then $\left(u_{1}^{*},u_{2}^{*}\right)$ is called a partial optimum of (BCP). A partial optimum $\left(u_{1}^{*},u_{2}^{*}\right)$ of (BCP) means that $u_{1}^{*}$ is an optimal solution to (BCP)($u_{2}^{*}$) and $u_{2}^{*}$ is an optimal solution to (BCP)($u_{1}^{*}$).

Next, let us give the equivalence of a partial optimum of (BCP) to a partial KKT point under some conditions.

Let $h,g_{i}:R^{n_{1}}\times R^{n_{2}}\to R,\;i\in I=\left\{1,2,\cdots ,m\right\}$ be biconvex and differentiable. Let $(u_{1}^{*},u_{2}^{*})\in U$. If there are $\alpha_{i}\left(i=1,2,\cdots ,m\right)$ such that:

(3) $$\begin{array}{c} \nabla h\left(u_{1}^{*},u_{2}^{*}\right)+\sum_{i=1}^{m}\alpha_{i}\nabla g_{i}\left(u_{1}^{*},u_{2}^{*}\right)=0, \end{array}$$

(4) $$\begin{array}{c} \alpha_{i}g_{i}\left(u_{1}^{*},u_{2}^{*}\right)=0,\alpha_{i}\geq 0,i=1,2,\cdots ,m, \end{array}$$

then $\left(u_{1}^{*},u_{2}^{*}\right)$ is a KKT point of (BCP).

Let $(u_{1}^{*},u_{2}^{*})\in U$. If there are $\alpha_{i}^{\left(1\right)},\alpha_{i}^{\left(2\right)}\left(i=1,2,\cdots ,m\right)$ such that:

(5) $$\begin{array}{c} \nabla_{u_{1}}h\left(u_{1}^{*},u_{2}^{*}\right)+\sum_{i=1}^{m}\alpha_{i}^{\left(1\right)}\nabla_{u_{1}}g_{i}\left(u_{1}^{*},u_{2}^{*}\right)=0, \end{array}$$

(6) $$\begin{array}{c} \nabla_{u_{2}}h\left(u_{1}^{*},u_{2}^{*}\right)+\sum_{i=1}^{m}\alpha_{i}^{\left(2\right)}\nabla_{u_{2}}g_{i}\left(u_{1}^{*},u_{2}^{*}\right)=0, \end{array}$$

(7) $$\begin{array}{c} \alpha_{i}^{\left(1\right)}g_{i}\left(u_{1}^{*},u_{2}^{*}\right)=0,\alpha_{i}^{\left(2\right)}g_{i}\left(u_{1}^{*},u_{2}^{*}\right)=0,\alpha_{i}^{1}\geq 0,\alpha_{i}^{2}\geq 0,i=1,2,\cdots ,m, \end{array}$$

then $\left(u_{1}^{*},u_{2}^{*}\right)$ is a partial KKT point of (BCP).

Let $(u_{1}^{*},u_{2}^{*})\in U$. The constraint of (BCP) is called a partial Slater constraint qualification at $\left(u_{1}^{*},u_{2}^{*}\right)$, if there is $\left({\bar{u}}_{1},{\bar{u}}_{2}\right)\in R^{n_{1}}\times R^{n_{2}}$ such that:

$$g_{i}\left(u_{1}^{*},{\bar{u}}_{2}\right)<0,g_{i}\left({\bar{u}}_{1},u_{2}^{*}\right)<0,i=1,2,\cdots ,m.$$

In fact, if the Slater constraint qualification is satisfied for a convex programming, an optimal solution of the convex programming is equal to a KKT condition. For biconvex programming, we have the results in [28] as follows.

**Theorem** **1.** Let $(u_{1}^{*},u_{2}^{*})\in u$. If (BCP) is satisfied with partial Slater constraint qualification at $\left(u_{1}^{*},u_{2}^{*}\right)$, then $\left(u_{1}^{*},u_{2}^{*}\right)$ is a partial optimum of (BCP) if and only if $\left(u_{1}^{*},u_{2}^{*}\right)$ is a partial KKT point of (BCP).

**Corollary** **1.** Let $(u_{1}^{*},u_{2}^{*})\in u$ be a partial optimum of (BCP). If (BCP) is satisfied with partial Slater constraint qualification at $\left(u_{1}^{*},u_{2}^{*}\right)$, then $\left(u_{1}^{*},u_{2}^{*}\right)$ is a KKT point of (BCP) if and only if (5), (6), and (7) hold with $\alpha_{i}^{1}=\alpha_{i}^{2},i=1,2,\cdots ,m$.

**Example** **1.** *Let the biconvex programming:*

$$\begin{array}{ccc} (BCP1)\;\min & & h(x,y)=xy\\ s.t. & & \;g_{1}\left(x,y\right)=1-x-y\leq 0, \end{array}$$

*where $h,g_{1}:R\times R\to R$. For ∀θ∈(0,1), it is clear that (x,y)=(θ,1−θ) is the partial KKT point and the partial optimum of (BCP1). If y=1−x, then $h\left(x,y\right)=x-x^{2}$. We have h(x,y)→−∞ as x→∞. Hence, a local optimal solution to (BCP1) cannot be solved, such as in $\left(x,y\right)=\left(\frac{1}{2},\frac{1}{2}\right)$.*

Example 1 means that if there is no optimal solution to biconvex programming, there may exist a partial KKT point or a partial optimum. It is obvious that an optimal solution to biconvex programming is the partial optimum. For biconvex programming, there is a partial optimum even if an optimal solution to biconvex programming is not obtained. Example 1 further indicates that the partial optimum is very important to biconvex programming.

Let ρ>0 be given. Consider the following optimization problem:

$$(\mathrm{BCP}\left(u_{2};\rho \right))\;\;\min\;\;H_{p}\left(u_{1},u_{2},\rho \right),\;\;s.t.\;u_{1}\in R^{n_{1}}.$$

where $u_{1}$ is a decision variable when $u_{2}$ is fixed, and

$$(\mathrm{BCP}\left(u_{1};\rho \right))\;\;\min\;\;H_{p}\left(u_{1},u_{2},\rho \right),\;\;s.t.\;u_{2}\in R^{n_{2}}.$$

where $u_{2}$ is a decision variable when $u_{1}$ is fixed.

**Definition** **2.** Let $\left({\bar{u}}_{1}^{*},{\bar{u}}_{2}^{*}\right)\in R^{n_{1}}\times R^{n_{2}}$. If:

(8) $$\begin{array}{c} H_{p}\left({\bar{u}}_{1}^{*},{\bar{u}}_{2}^{*},\rho \right)\leq H_{p}\left(u_{1},{\bar{u}}_{2}^{*},\rho \right),\forall u_{1}\in R^{n_{1}}, \end{array}$$

(9) $$\begin{array}{c} H_{p}\left({\bar{u}}_{1}^{*},{\bar{u}}_{2}^{*},\rho \right)\leq H_{p}\left({\bar{u}}_{1}^{*},u_{2},\rho \right),\forall u_{2}\in R^{n_{2}}, \end{array}$$

then $\left({\bar{u}}_{1}^{*},{\bar{u}}_{2}^{*}\right)$ is called a partial optimum of $H_{p}\left(u_{1},u_{2},\rho \right)$.

Next, we define the concept of a partially exact penalty function for biconvex programming as follows.

**Definition** **3.** (1) Let $\left(u_{1}^{*},u_{2}^{*}\right)$ be a partial optimum of (BCP). If there is a $\rho^{\prime }$ such that $\left(u_{1}^{*},u_{2}^{*}\right)$ is a partial optimum of $H_{p}\left(u_{1},u_{2},\rho \right)$ for $\forall \rho >\rho^{\prime }$, then $H_{p}\left(u_{1},u_{2},\rho \right)$ is called a partially exact penalty function.

(2) Let $\left(u_{1}^{*},u_{2}^{*}\right)$ be a partial optimum of $H_{p}\left(u_{1},u_{2},\rho \right)$. If $\left(u_{1}^{*},u_{2}^{*}\right)$ of $H_{p}\left(u_{1},u_{2},\rho \right)$ is a partial optimum of (BCP), then ρ is called a partially exact value of the penalty parameter.

Example 2 below shows the partial exactness of a penalty function under different *p* values.

**Example** **2.** *Let the biconvex programming:*

$$\begin{array}{ccc} (BCP2)\;\min & & h(x,y)=xy+x+y\\ s.t. & & \;x^{2}+y^{2}\leq 0. \end{array}$$

It is clear that (0,0) is a partial optimum of (BCP2). The penalty function for (BCP2) is defined by:

$$H_{p}\left(x,y;\rho \right)=xy+x+y+\rho \max{\left\{x^{2}+y^{2},0\right\}}^{p}.$$

When p=1, a partial optimum of $\min_{x,y\in R}H_{1}\left(x,y;\rho \right)$ is $\left(x\left(\rho \right),y\left(\rho \right)\right)=\left(-\frac{1}{2\rho +1},-\frac{1}{2\rho +1}\right)$. Letting ρ→+∞, we have (x(ρ),y(ρ))→(0,0). Hence, $H_{1}\left(x,y,\rho \right)$ is not a partially exact penalty function. When p=0.5, we have:

$$H_{0.5}\left(x,y;\rho \right)=xy+x+y+\rho \sqrt{x^{2}+y^{2}}.$$

A partial optimum of $\min_{x,y\in R}H_{0.5}\left(x,y;\rho \right)$ is (0,0) for ρ≥0. Hence, $H_{0.5}\left(x,y,\rho \right)$ is a partially exact penalty function for ρ≥1. Example 2 means that the partial exactness of penalty function depends on the parameter *p*.

**Example** **3.** *Let the biconvex programming:*

$$\begin{array}{ccc} (BCP3)\;\min & & h(x,y)=xy\\ s.t. & & \;xy\geq 1. \end{array}$$

It is easy to verify that $\left(x^{*},y^{*}\right)={\left(\theta ,\frac{1}{\theta }\right)}^{T}$ is an optimal solution to (BCP3) for any θ≠0. Thus, $\left(x^{*},y^{*}\right)={\left(\theta ,\frac{1}{\theta }\right)}^{T}$ is a partial optimum of (BCP3) too. The example illustrates that all partial optimums of (BCP) may be optimal solutions.

$\left(x^{*},y^{*}\right)={\left(1,1\right)}^{T}$ is a partial optimum of (BCP3). The square penalty function for (BCP3) is defined by:

$$H_{2}\left(x,y;\rho \right)=xy+\rho \max{\left\{0,1-xy\right\}}^{2}.$$

It is easy to check that $\left(x^{*},y^{*}\right)=\left(\frac{1}{\delta }\left(1-\frac{1}{2\rho }\right),\delta \right)$ is a partial optimum of $H_{2}\left(x,y,\rho \right)$ for δ≠0 and ρ≥1. It is easy to check that $H_{2}\left(x,y;\rho \right)$ is not partially exact.

For 0<p≤1, a penalty function for (BCP3) is defined by:

$$H_{p}\left(x,y;\rho \right)=xy+\rho \max{\left\{0,1-xy\right\}}^{p}.$$

We have that $\left(x^{*},y^{*}\right)=\left(1,1\right)$ is a partial optimum of $H_{p}\left(x,y;\rho \right)$ for ρ>1. It is easy to check that $H_{p}\left(x,y;\rho \right)$ is partially exact for ρ>1. We easily check that (1,1) is a KKT point.

Example 3 illustrates that the partially exact penalty function for a partial optimum can be as good as traditional exact penalty functions.

We prove the similarity of the partially exact penalty result to [28].

**Theorem** **2.** Let $h\left(u_{1},u_{2}\right)\geq 0,\forall \left(u_{1},u_{2}\right)\in R^{n_{1}}\times R^{n_{2}}$. Let $(u_{1}^{*},u_{2}^{*})\in U$ be a partial optimum of (BCP). If $\left(u_{1}^{*},u_{2}^{*}\right)$ is a KKT partial point of (BCP), i.e., there are $\alpha_{i}^{\left(1\right)},\alpha_{i}^{\left(2\right)}\left(i=1,2,\cdots ,m\right)$ such that (5), (6), and (7) are true, then $H_{p}\left(u_{1},u_{2},\rho \right)$ is a partially exact penalty function for $\rho >\rho^{*}$, where:

$$\rho^{*}=\max\left\{h\left(u_{1}^{*},u_{2}^{*}\right),\alpha_{i}^{\left(1\right)},\alpha_{i}^{\left(2\right)}|i=1,2,\cdots ,m\right\}.$$

**Proof.**  If $\left(u_{1}^{*},u_{2}^{*}\right)$ is a partial KKT point of (BCP), there are $\alpha_{i}^{\left(1\right)}$ and $\alpha_{i}^{\left(2\right)}\left(i=1,2,\cdots ,m\right)$ such that (5), (6) and (7) are true. Because $h\left(u_{1},u_{2}\right),g_{i}\left(u_{1},u_{2}\right)\left(\forall i\in I\right)$ are biconvex functions for $\forall u_{1}\in R^{n_{1}}$ and $\forall u_{2}\in R^{n_{2}}$, we have:

(10) $$\begin{array}{ccc} h(u_{1},u_{2}^{*}) & \geq & h\left(u_{1}^{*},u_{2}^{*}\right)+\nabla_{u_{1}}h{\left(u_{1}^{*},u_{2}^{*}\right)}^{T}\left(u_{1}-u_{1}^{*}\right), \end{array}$$

(11) $$\begin{array}{ccc} g_{i}\left(u_{1},u_{2}^{*}\right) & \geq & g_{i}\left(u_{1}^{*},u_{2}^{*}\right)+\nabla_{u_{1}}g_{i}{\left(u_{1}^{*},u_{2}^{*}\right)}^{T}\left(u_{1}-u_{1}^{*}\right),i\in I, \end{array}$$

(12) $$\begin{array}{ccc} h(u_{1}^{*},u_{2}) & \geq & h\left(u_{1}^{*},u_{2}^{*}\right)+\nabla_{u_{2}}h{\left(u_{1}^{*},u_{2}^{*}\right)}^{T}\left(u_{2}-u_{2}^{*}\right), \end{array}$$

(13) $$\begin{array}{ccc} g_{i}\left(u_{1}^{*},u_{2}\right) & \geq & g_{i}\left(u_{1}^{*},u_{2}^{*}\right)+\nabla_{u_{2}}g_{i}{\left(u_{1}^{*},u_{2}^{*}\right)}^{T}\left(u_{2}-u_{2}^{*}\right),i\in I. \end{array}$$

By (5), (7), (10), and (11), we have:

(14) $$\begin{array}{ccc} h(u_{1},u_{2}^{*}) & \geq & h\left(u_{1}^{*},u_{2}^{*}\right)-\sum_{i=1}^{m}\alpha_{i}^{\left(1\right)}\nabla_{u_{1}}g_{i}{\left(u_{1}^{*},u_{2}^{*}\right)}^{T}\left(u_{1}-u_{1}^{*}\right)\\ & \geq & h\left(u_{1}^{*},u_{2}^{*}\right)+\sum_{i=1}^{m}\alpha_{i}^{\left(1\right)}\left(g_{i}\left(u_{1}^{*},u_{2}^{*}\right)-g_{i}\left(u_{1},u_{2}^{*}\right)\right)\\ & = & h\left(u_{1}^{*},u_{2}^{*}\right)-\sum_{i=1}^{m}\alpha_{i}^{\left(1\right)}g_{i}\left(u_{1},u_{2}^{*}\right). \end{array}$$

Let $P\left(t\right)=\max{\left\{t,0\right\}}^{p},t\in R$, and 0<p≤1. Let us take $\rho >\rho^{*}$ with $\rho^{*}=\max\{h\left(u_{1}^{*},u_{2}^{*}\right),$$\alpha_{i}^{\left(1\right)},\alpha_{i}^{\left(2\right)}\left|i=1,2,\cdots ,m\right\}.$ If there is a $g_{i}\left(u_{1},u_{2}^{*}\right)>1$, then $P(g_{i}\left(u_{1},u_{2}^{*}\right))>1$. We have:

$$\begin{array}{c} h\left(u_{1},u_{2}^{*}\right)+\rho \sum_{i=1}^{m}P\left(g_{i}\left(u_{1},u_{2}^{*}\right)\right)\geq \rho^{*}\geq h\left(u_{1}^{*},u_{2}^{*}\right), \end{array}$$

i.e.,

$$H_{p}\left(u_{1},u_{2}^{*},\rho \right)\geq H_{p}\left(u_{1}^{*},u_{2}^{*},\rho \right).$$

Otherwise, if $g_{i}\left(u_{1},u_{2}^{*}\right)\leq 1$ for ∀i∈I, from (14) we have:

$$\begin{array}{ccc} h\left(u_{1},u_{2}^{*}\right)+\rho \sum_{i=1}^{m}P\left(g_{i}\left(u_{1},u_{2}^{*}\right)\right) & \geq & h(u_{1}^{*},u_{2}^{*})\\ & & +\sum_{i=1}^{m}\left(\rho \max{\left\{g_{i}\left(u_{1},u_{2}^{*}\right),0\right\}}^{p}-\alpha_{i}^{\left(1\right)}g_{i}\left(u_{1},u_{2}^{*}\right)\right). \end{array}$$

Hence, for $\rho >\rho^{*}$, we have:

(15) $$\begin{array}{c} H_{p}\left(u_{1}^{*},u_{2}^{*},\rho \right)\leq H_{p}\left(u_{1},u_{2}^{*},\rho \right). \end{array}$$

Similarly, for $\rho >\rho^{*}$, we have:

$$\begin{array}{c} H_{p}\left(u_{1}^{*},u_{2}^{*},\rho \right)\leq H_{p}\left(u_{1}^{*},u_{2},\rho \right). \end{array}$$

By Definition 2, we have that $H_{p}\left(u_{1},u_{2},\rho \right)$ is a partially exact penalty function for $\rho >\rho^{*}$.

□

**Note.** If there is a number *A* such that $h(u_{1},u_{2})\geq A$ for any $\left(u_{1},u_{2}\right)\in R^{n_{1}}\times R^{n_{2}}$, then the conclusion of Theorem 2 holds too. If we let $h_{1}\left(u_{1},u_{2}\right)=h\left(u_{1},u_{2}\right)-A$, we have $h_{1}\left(u_{1},u_{2}\right)\geq 0$ on $R^{n_{1}}\times R^{n_{2}}$. It is clear that the problem

$$\begin{array}{ccc} {\left(\mathrm{BCP}\right)}_{1}\; & \min\; & h_{1}\left(u_{1},u_{2}\right)\\ & s.t.\; & g_{i}\left(u_{1},u_{2}\right)\leq 0,\;i=1,2,\cdots ,m, \end{array}$$

is equal to the problem (BCP). Particularly, when p=1, we have the following conclusion.

**Theorem** **3.** Let $(u_{1}^{*},u_{2}^{*})\in U$ be a partial optimum of (BCP). If $\left(u_{1}^{*},u_{2}^{*}\right)$ is a KKT partial point of (BCP), i.e., there are $\alpha_{i}^{\left(1\right)}$ and $\alpha_{i}^{\left(2\right)}\left(i=1,2,\cdots ,m\right)$ such that (5), (6), and (7) are true, then $H_{1}\left(u_{1},u_{2},\rho \right)$ is a partially exact penalty function with p=1 for $\rho >\rho^{*}$, where:

$$\rho^{*}=\max\left\{\alpha_{i}^{\left(1\right)},\alpha_{i}^{\left(2\right)}|i=1,2,\cdots ,m\right\}.$$

Theorem 3 is consistent with Theorem 2 in [28].

Similar to that for a constrained penalty function presented in [25,26], the concept of stability for a penalty function of (BCP) is defined. Let $s_{1}=\left(s_{1}^{1},s_{2}^{1},\cdots ,s_{m}^{1}\right)$ and:

$$U\left(u_{2},s_{1}\right)=\left\{u_{1}\in R^{n_{1}}|g_{i}\left(u_{1},u_{2}\right)\leq s_{i}^{1},\;i\in I\right\}.$$

When $u_{2}$ is fixed, define a perturbed problem:

$$\begin{array}{ccc} \left(\mathrm{BCP}\right)\left(u_{2},s_{1}\right)\; & \min\; & h(u_{1},u_{2})\\ & s.t.\; & u_{1}\in U\left(u_{2},s_{1}\right). \end{array}$$

Let $s_{2}=\left(s_{1}^{2},s_{2}^{2},\cdots ,s_{m}^{2}\right)$ and:

$$U\left(u_{1},s_{2}\right)=\left\{u_{2}\in R^{n_{2}}|g_{i}\left(u_{1},u_{2}\right)\leq s_{i}^{2},\;i\in I\right\}.$$

When $u_{1}$ is fixed, define a perturbed problem:

$$\begin{array}{ccc} \left(\mathrm{BCP}\right)\left(u_{1},s_{2}\right)\; & \min\; & h(u_{1},u_{2})\\ & s.t.\; & u_{2}\in U\left(u_{1},s_{2}\right). \end{array}$$

**Definition** **4.** Let $\left(u_{1}^{*},u_{2}^{*}\right)$ be a partial optimum of (BCP), and $u_{1s_{1}}^{*}$ and $u_{2s_{2}}^{*}$ be optimal solutions to (BCP)($u_{2}^{*},s_{1}$) and (BCP)($u_{1}^{*},s_{2}$), respectively, for any $s_{1},s_{2}\in R^{m}$. If there is a $\rho^{\prime }>0$ such that:

(16) $$\begin{array}{c} h\left(u_{1}^{*},u_{2}^{*}\right)-h\left(u_{1s_{1}}^{*},u_{2}^{*}\right)\leq \rho \left|s_{1}\right|,\;\;\;\;\forall \rho >\rho^{\prime }, \end{array}$$

(17) $$\begin{array}{c} h\left(u_{1}^{*},u_{2}^{*}\right)-h\left(u_{1}^{*},u_{2s_{2}}^{*}\right)\leq \rho \left|s_{2}\right|,\;\;\;\;\forall \rho >\rho^{\prime }, \end{array}$$

where $|s_{1}|=\sum_{i=1}^{m}P\left(s_{i}^{1}\right)$ and $|s_{2}|=\sum_{i=1}^{m}P\left(s_{i}^{2}\right)$, then $H_{p}\left(u_{1},u_{2},\rho \right)$ is partially stable at $\left(u_{1}^{*},u_{2}^{*}\right)$. Furthermore, if there are a γ>0 and a $\rho^{\prime }>0$ such that (16) and (17) hold for $|s_{1}|\leq \gamma$ and $|s_{2}|\leq \gamma$, then $H_{p}\left(u_{1},u_{2},\rho \right)$ is partially locally stable at $\left(u_{1}^{*},u_{2}^{*}\right)$.

**Theorem** **4.** Let $\left(u_{1}^{*},u_{2}^{*}\right)$ be a partial optimum of (BCP). If $H_{p}\left(u_{1},u_{2},\rho \right)$ is partially stable, then $H_{p}\left(u_{1},u_{2},\rho \right)$ is a partially exact penalty function at $\left(u_{1}^{*},u_{2}^{*}\right)$.

**Proof.**  Let us prove that $H_{p}\left(u_{1},u_{2},\rho \right)$ is a partially exact penalty function when $H_{p}\left(u_{1},u_{2},\rho \right)$ is partially stable at $\left(u_{1}^{*},u_{2}^{*}\right)$. Suppose that $H_{p}\left(u_{1},u_{2},\rho \right)$ is not a partially exact penalty function. According to the definition of partial stability, for any $s_{1},s_{2}$, we obtain that there is a $\rho^{\prime }$ satisfying that:

(18) $$\begin{array}{c} h\left(u_{1}^{*},u_{2}^{*}\right)-h\left(u_{1s_{1}}^{*},u_{2}^{*}\right)\leq \rho \left|s_{1}\right|,\forall \rho >\rho^{\prime }, \end{array}$$

(19) $$\begin{array}{c} h\left(u_{1}^{*},u_{2}^{*}\right)-h\left(u_{2}^{*},u_{2s_{2}}^{*}\right)\leq \rho \left|s_{2}\right|,\forall \rho >\rho^{\prime }. \end{array}$$

Then, there always exists some $\rho >\rho^{\prime }$ such that $\left(u_{1}^{*},u_{2}^{*}\right)$ is not a partial optimum of $H_{p}\left(u_{1},u_{2},\rho \right)$, i.e., there is some $\left(u_{1}^{\prime },u_{2}^{\prime }\right)$ such that:

$$H_{p}\left(u_{1}^{\prime },u_{2}^{*},\rho \right)<H_{p}\left(u_{1}^{*},u_{2}^{*},\rho \right)=h\left(u_{1}^{*},u_{2}^{*}\right),$$

$$H_{p}\left(u_{1}^{*},u_{2}^{\prime },\rho \right)<H_{p}\left(u_{1}^{*},u_{2}^{*},\rho \right)=h\left(u_{1}^{*},u_{2}^{*}\right).$$

Thus,

$$h\left(u_{1}^{\prime },u_{2}^{*}\right)+\rho \sum_{i\in I}P\left(g_{i}\left(u_{1}^{\prime },u_{2}^{*}\right)\right)<h\left(u_{1}^{*},u_{2}^{*}\right),$$

$$h\left(u_{1}^{*},u_{2}^{\prime }\right)+\rho \sum_{i\in I}P\left(g_{i}\left(u_{1}^{*},u_{2}^{\prime }\right)\right)<h\left(u_{1}^{*},u_{2}^{*}\right).$$

Suppose that $u_{1}^{\prime }\in U\left(u_{2}^{*}\right),u_{2}^{\prime }\in U\left(u_{1}^{*}\right)$. We have:

$$h\left(u_{1}^{\prime },u_{2}^{*}\right)<h\left(u_{1}^{*},u_{2}^{*}\right),$$

$$h\left(u_{1}^{*},u_{2}^{\prime }\right)<h\left(u_{1}^{*},u_{2}^{*}\right).$$

This implies that $h\left(u_{1}^{\prime },u_{2}^{*}\right)<h\left(u_{1}^{*},u_{2}^{*}\right)<h\left(u_{1}^{\prime },u_{2}^{*}\right)$ and $h\left(u_{1}^{*},u_{2}^{\prime }\right)<h\left(u_{1}^{*},u_{2}^{*}\right)<h\left(u_{1}^{*},u_{2}^{\prime }\right)$, which shows that $\left(u_{1}^{*},u_{2}^{*}\right)$ is not a partial optimum of (BCP). A contradiction occurs. Hence, $u_{1}^{\prime }\in U\left(u_{2}^{*}\right)$ and $u_{2}^{\prime }\in U\left(u_{1}^{*}\right)$ do not hold, and $\sum_{i\in I}P\left(g_{i}\left(u_{1}^{\prime },u_{2}^{*}\right)\right)>0$ or $\sum_{i\in I}P\left(g_{i}\left(u_{1}^{*},u_{2}^{\prime }\right)\right)>0$.

Let $s_{1}^{\prime }={\left(s_{1}^{1^{\prime }},s_{2}^{1^{\prime }},\ldots ,s_{m}^{1^{\prime }}\right)}^{⊤}$ with $s_{i}^{1^{\prime }}=g_{i}\left(u_{1}^{\prime },u_{2}^{*}\right)$ and $s_{2^{\prime }}={\left(s_{1}^{2^{\prime }},s_{2}^{2^{\prime }},\ldots ,s_{m}^{2^{\prime }}\right)}^{⊤}$ with $s_{i}^{2}=g_{i}\left(u_{1}^{*},u_{2}^{\prime }\right)$, i=1,2,⋯,m, and $u_{1s_{1}^{\prime }}^{*}$ and $u_{2s_{2}^{\prime }}^{*}$ be optimal solutions to (BCP)($u_{2}^{*},s_{1}^{\prime }$) and (BCP)($u_{1}^{*},s_{2}^{\prime }$), respectively. Then, $h\left(u_{1s_{1}^{\prime }}^{*},u_{2}^{*}\right)\leq h\left(u_{1}^{\prime },u_{2}^{*}\right)$ and $h\left(u_{1}^{*},u_{2s_{2}^{\prime }}^{*}\right)\leq h\left(u_{1}^{*},u_{2}^{\prime }\right)$. Thus,

$$h\left(u_{1s_{1}^{\prime }}^{*},u_{2}^{*}\right)\leq h\left(u_{1}^{\prime },u_{2}^{*}\right),$$

$$h\left(u_{1}^{*},u_{2s_{2}^{\prime }}^{*}\right)\leq h\left(u_{1}^{*},x_{2}^{\prime }\right).$$

Therefore,

$$\begin{array}{ccc} h\left(u_{1s_{1}^{\prime }}^{*},u_{2}^{*}\right)+\rho \sum_{i\in I}P\left(s_{i}^{1^{\prime }}\right) & \leq & h\left(u_{1}^{\prime },u_{2}^{*}\right)+\rho \sum_{i\in I}P\left(s_{i}^{1^{\prime }}\right)\\ & = & H_{p}\left(u_{1}^{\prime },u_{2}^{*},\rho \right)<h\left(u_{1}^{*},u_{2}^{*}\right), \end{array}$$

and:

$$\begin{array}{ccc} h\left(u_{1}^{*},u_{2s_{2}^{\prime }}^{*}\right)+\rho \sum_{i\in I}P\left(s_{i}^{2^{\prime }}\right) & \leq & h\left(u_{1}^{*},u_{2}^{\prime }\right)+\rho \sum_{i\in I}P\left(s_{i}^{2^{\prime }}\right)\\ & = & H_{p}\left(u_{1}^{*},u_{2}^{\prime },\rho \right)<h\left(u_{1}^{*},u_{2}^{*}\right), \end{array}$$

which shows that:

$$h\left(u_{1}^{*},u_{2}^{*}\right)-h\left(u_{1}^{*},u_{2s_{2}^{\prime }}^{*}\right)>\rho \left|s_{1}^{\prime }\right|,$$

$$h\left(u_{1}^{*},u_{2}^{*}\right)-h\left(u_{1s_{1}^{\prime }}^{*},u_{2}^{*}\right)>\rho \left|s_{2}^{\prime }\right|,$$

where $|s_{1}^{\prime }|=\sum_{i\in I}P\left(s_{i}^{1^{\prime }}\right)$ and $|s_{2}^{\prime }|=\sum_{i\in I}P\left(s_{i}^{2^{\prime }}\right)$. These inequalities contradict (18) and (19). Hence, that $H_{p}\left(x,y,\rho \right)$ is not partially stable yields a contradiction with the assumption, which proves that $H_{p}\left(x,y,\rho \right)$ is a partially exact penalty function. □

**Theorem** **5.** Let $\left(u_{1}^{*},u_{2}^{*}\right)$ be a partial optimum of (BCP). If $H_{p}\left(u_{1},u_{2},\rho \right)$ is a partially exact penalty function at $\left(u_{1}^{*},u_{2}^{*}\right)$, then $H_{p}\left(u_{1},u_{2},\rho \right)$ is partially locally stable. In particular, for p=1, $H_{1}\left(u_{1},u_{2},\rho \right)$ is partially stable.

**Proof.**  Let us prove that $H_{p}\left(x,y,\rho \right)$ is partially locally stable when $H_{p}\left(x,y,\rho \right)$ is a partially exact penalty function. According to the definition of a partially exact penalty function, if $\left(u_{1}^{*},u_{2}^{*}\right)$ is a partial optimum of (BCP), there always exist some $\rho >\rho^{\prime }$ such that:

(20) $$\begin{array}{c} H_{p}\left(u_{1}^{*},u_{2}^{*},\rho \right)\leq H_{p}\left(u_{1},u_{2}^{*},\rho \right),\forall u_{1}\in R^{n_{1}}, \end{array}$$

(21) $$\begin{array}{c} H_{p}\left(u_{1}^{*},u_{2}^{*},\rho \right)\leq H_{p}\left(u_{1}^{*},u_{2},\rho \right),\forall u_{2}\in R^{n_{2}}. \end{array}$$

Let $u_{1s_{1}}^{*}$ and $u_{2s_{2}}^{*}$ be optimal solutions to (BCP)($u_{2}^{*},s_{1}$) and (BCP)($u_{1}^{*},s_{2}$), respectively, for any $s_{1},s_{2}\in R^{m}$. By (20), and (21), we have:

(22) $$\begin{array}{c} h\left(u_{1}^{*},u_{2}^{*}\right)=H_{p}\left(u_{1}^{*},u_{2}^{*},\rho \right)\leq H_{p}\left(u_{1s_{1}}^{*},u_{2}^{*},\rho \right), \end{array}$$

(23) $$\begin{array}{c} h\left(u_{1}^{*},u_{2}^{*}\right)=H_{p}\left(u_{1}^{*},u_{2}^{*},\rho \right)\leq H_{p}\left(u_{1}^{*},u_{2s_{2}}^{*},\rho \right). \end{array}$$

It is clear that for $s_{i}^{1}\leq 1,s_{i}^{2}\leq 1\left(i\in I\right)$, we have:

(24) $$\begin{array}{c} P\left(g_{i}\left(u_{1s_{1}}^{*},u_{2}^{*}\right)\right)\leq P\left(s_{i}^{1}\right),\;P\left(g_{i}\left(u_{1}^{*},u_{2s_{2}}^{*}\right)\right)\leq P\left(s_{i}^{2}\right),\;i\in I. \end{array}$$

From (22), (23), and (24), for $|s_{1}|\leq m$ and $|s_{2}|\leq m$:

$$h\left(u_{1}^{*},u_{2}^{*}\right)-h\left(u_{1s_{1}}^{*},u_{2}^{*}\right)\leq \rho \left|s_{1}\right|,\;\;\;\;\forall \rho >\rho^{\prime },$$

$$h\left(u_{1}^{*},u_{2}^{*}\right)-h\left(u_{1}^{*},u_{2s_{2}}^{*}\right)\leq \rho \left|s_{2}\right|,\;\;\;\;\forall \rho >\rho^{\prime }.$$

It follows from the definition that $H_{p}\left(x,y,\rho \right)$ is partially locally stable. □

Theorem 4 and Theorem 5 mean that the stability condition is sufficient for the partial exact penalty function, but the necessary condition of the partial exact penalty function is partially locally stable, as shown in the following example.

**Example** **4.** *Let the perturbed problem (BCP3), and its (BCP3($x,s_{1}$)) and (BCP3($y,s_{2}$)):*

$$\begin{array}{ccc} \left(BCP3\right)\left(x,s_{1}\right)\;\min_{y} & & h(x,y)=xy\\ s.t. & & \;1-xy\leq s_{1},\\ \left(BCP3\right)\left(y,s_{2}\right)\;\min_{x} & & h(x,y)=xy\\ s.t. & & \;1-xy\leq s_{2}. \end{array}$$

It is clear that $\left(x^{*},y^{*}\right)=\left(1,1\right)$ is a partial optimum of (MCP3) and its objective function value is 1. $x_{s_{1}}^{*}=1-s_{1}$ and $y_{s_{2}}^{*}=1-s_{2}$ are optimal solutions to (BCP3)($1,s_{1}$) and (BCP3)($1,s_{2}$), respectively, for any $s_{1},s_{2}\in R$. Let $P\left(t\right)=\max{\left\{t,0\right\}}^{p}$ and $s_{1},s_{2}\leq 1$ with 0<p<1. For ρ>1,

$$h\left(x^{*},y^{*}\right)-h\left(x^{*},y_{s_{1}}^{*}\right)=1-\left(1-s_{1}\right)\leq \rho P\left(s_{1}\right),$$

$$h\left(x^{*},y^{*}\right)-h\left(x_{s_{1}}^{*},y^{*}\right)=1-\left(1-s_{2}\right)\leq \rho P\left(s_{2}\right),$$

then $H_{p}\left(x,y,\rho \right)$ is partially locally stable. When p=1, $H_{1}\left(x,y,\rho \right)$ is partially stable.

## 3. Partial Optimum Penalty Function Algorithm for (BCP)

Now, we present an algorithm to solve a partial optimum of (BCP) by solving the penalty function problem BCP(ρ) as follows.

$$\begin{array}{ccc} BCP(\rho ) & \min & H_{p}\left(u_{1},u_{2},\rho \right)\;\;s.t.\left(u_{1},u_{2}\right)\in R^{n_{1}}\times R^{n_{2}}. \end{array}$$

Based on the above results, the Partial Optimum Penalty Function Algorithm (Algorithm 1) was designed to compute a partial optimum of (BCP). 

We prove the convergence of the Algorithm 1 in Theorem 6. Let:

$$S\left(L,h\right)=\{\left(u_{1},u_{2}\right)|L\geq h\left(u_{1},u_{2}\right),\}$$

be a level set. If for any given L>0, S(L,h) is bounded, then S(L,h) is called bounded.
**Algorithm 1:** POPFA Algorithm**Step 1:** Choose $\rho_{1}>0,N>1,0<p\leq 1$ and k=1. **Step 2:** Solve $\left(u_{1}^{k},u_{2}^{k}\right)$ to be a partial optimum of$$\min H_{p}\left(u_{1},u_{2},\rho_{k}\right)\;\;s.t.\left(u_{1},u_{2}\right)\in R^{n_{1}}\times R^{n_{2}}.$$**Step 3:** If $\left(u_{1}^{k},u_{2}^{k}\right)$ is feasible to (BCP), stop and $\left(u_{1}^{k},u_{2}^{k}\right)$ is a partial optimum of (BCP). Otherwise, $\rho_{k+1}=N\rho_{k},k=:k+1$ and go to Step 2.

**Theorem** **6.** *Let $\left\{\left(u_{1}^{k},u_{2}^{k}\right)\right\}$ be the sequence generated by the Algorithm 1 and h(·) be continuous.*

*(i) If $\left\{\left(u_{1}^{k},u_{2}^{k}\right)\right\}\left(k=1,2,\cdots ,\bar{k}\right)$ is a finite sequence (i.e., the Algorithm 1 stops at the $\bar{k}$-th iteration), then $\left(u_{1}^{\bar{k}},u_{2}^{\bar{k}}\right)$ is a partial optimum of (BCP).*

(ii) Let $\left\{\left(u_{1}^{k},u_{2}^{k}\right)\right\}$ be an infinite sequence, sequence $\left\{H_{p}\left(u_{1}^{k},u_{2}^{k},\rho_{k}\right)\right\}$ be bounded and the level set S(L,h) be bounded. Then $\left\{\left(u_{1}^{k},u_{2}^{k}\right)\right\}$ is bounded and any limit point $\left(u_{1}^{*},u_{2}^{*}\right)$ of it is a partial optimum of (BCP).

**Proof.**  (i) The conclusion is clear.

(ii) By the Algorithm 1, since $\left\{H_{p}\left(u_{1}^{k},u_{2}^{k},\rho_{k}\right)\right\}$ is bounded as k→+∞, there must be some L>0 such that:

(25) $$\begin{array}{ccc} L & > & H_{p}\left(u_{1}^{k},u_{2}^{k},\rho_{k}\right)\\ & \geq & h\left(u_{1}^{k},u_{2}^{k}\right)+\rho_{k}\sum_{i=1}^{m}P\left(g_{i}\left(u_{1}^{k},u_{2}^{k}\right)\right)\\ & \geq & h(u_{1}^{k},u_{2}^{k}). \end{array}$$

Since *h* is continuous and the level set S(L,h) is bounded and closed, $\left\{\left(u_{1}^{k},u_{2}^{k}\right)\right\}$ and $\left\{h\left(u_{1}^{k},u_{2}^{k}\right)\right\}$ are bounded. Without loss of generality, suppose $\left(u_{1}^{k},u_{2}^{k}\right)\to \left(u_{1}^{*},u_{2}^{*}\right)$. Hence, there is an A>0 such that $h(u_{1}^{k},u_{2}^{k})>-A$. From (25), we have:

$$\begin{array}{c} \sum_{i=1}^{m}P\left(g_{i}\left(u_{1}^{k},u_{2}^{k}\right)\right)\leq \frac{1}{\rho_{k}}\left(L-h\left(u_{1}^{k},u_{2}^{k}\right)\right)<\frac{L+A}{\rho_{k}}. \end{array}$$

We have $\sum_{i=1}^{m}P\left(g_{i}\left(u_{1}^{k},u_{2}^{k}\right)\right)\to 0$ as $\rho_{k}\to +\infty$. Hence, $\left(u_{1}^{*},u_{2}^{*}\right)$ is a feasible solution to (BCP).

Let any ${\bar{u}}_{1}\in U\left(u_{2}^{*}\right)$ and ${\bar{u}}_{2}\in U\left(u_{1}^{*}\right)$. Since $\left(u_{1}^{k},u_{2}^{k}\right)$ is a partial optimum of

$$\min H_{p}\left(u_{1},u_{2},\rho_{k}\right)\;\;s.t.\left(u_{1},u_{2}\right)\in R^{n_{1}}\times R^{n_{2}},$$

we have:

$$h\left(u_{1}^{k},u_{2}^{k}\right)\leq H_{p}\left(u_{1}^{k},u_{2}^{k},\rho_{k}\right)\leq H_{p}\left({\bar{u}}_{1},u_{2}^{k},\rho_{k}\right),$$

$$h\left(u_{1}^{k},u_{2}^{k}\right)\leq H_{p}\left(u_{1}^{k},u_{2}^{k},\rho_{k}\right)\leq H_{p}\left(u_{1}^{k},{\bar{u}}_{2},\rho_{k}\right).$$

Let k→+∞, and the above inequations are:

$$h\left(u_{1}^{*},u_{2}^{*}\right)\leq h\left({\bar{u}}_{1},u_{2}^{*}\right),$$

$$h\left(u_{1}^{*},u_{2}^{*}\right)\leq h\left(u_{1}^{*},{\bar{u}}_{2}\right).$$

Hence, $\left(u_{1}^{*},u_{2}^{*}\right)$ is a partial optimum of (BCP). □

Theorem 6 means that the Algorithm 1 has good convergence in theory. From Theorem 4, when the penalty function is partially stable, the Algorithm 1 solves a single unconstrained optimization problem for the smaller penalty parameter ρ. Since the penalty function $H_{p}\left(u_{1},u_{2},\rho \right)$ is nonsmooth for 0<p<1, it is necessary to smooth the constrained nonsmooth term to design an effective algorithm. Therefore, the smoothing algorithm of a partial exact penalty function is worthy of further study. In fact, for p=1, we have published a paper regarding a smoothing partially exact penalty function algorithm [30], where two numerical examples show the proposed algorithm is effective for biconvex programming.

## 4. Conclusions

In this paper, we studied the partial optimum solution to biconvex programming using the penalty function, which is partially exact. The form of this penalty function is more general than that in [28]. We proved that the partial exactness of the penalty function for biconvex programming is equivalent to the partial KKT condition, and we proved that the partial exactness of the penalty function for biconvex programming is equivalent to partially local stability. Based on the penalty function, the Algorithm 1 was theoretically presented to solve a partial optimum solution to biconvex programming. The convergence of the algorithm was also proven. The Algorithm 1 may solve a partial optimum solution to biconvex programming under the smaller penalty parameter. In the future, we may study the smoothing problem of the partial exact penalty function and its algorithm.
